# Orexin Receptor-1 in the Rostral Ventrolateral Medulla Mediates the Antihypertensive Effects of Electroacupuncture

**DOI:** 10.3389/fnins.2019.00282

**Published:** 2019-03-26

**Authors:** Ying-Ying Tan, Ling Fang, Fan-Rong Yao, Dong-Yuan Cao, Qi Zhang

**Affiliations:** ^1^Shaanxi Key Laboratory of Chinese Medicine Encephalopathy, Shaanxi University of Chinese Medicine, Xianyang, China; ^2^Department of Biochemistry and Molecular Biology, Brody School of Medicine, East Carolina University, Greenville, NC, United States; ^3^Key Laboratory of Shaanxi Province for Craniofacial Precision Medicine Research, Research Center of Stomatology, Xi’an Jiaotong University College of Stomatology, Xi’an, China

**Keywords:** hypertension, electroacupuncture, orexin, rostral ventrolateral medulla, sympathetic

## Abstract

Electroacupuncture (EA) has been used to treat numerous diseases, including hypertension. This study aimed to investigate the long-term effect and underlying mechanisms of EA stimulation at the LI11 point on the hypertension and sympathetic nerve activity in two-kidney, one-clip (2K1C) hypertensive rats. EA (0.1–0.4 mA, 2 and 15 Hz) was applied to the acupoints LI11 overlying the deep radial nerve once a day for 6 weeks. The mean arterial pressure (MAP) and heart rate (HR) were determined by radiotelemetry, and the sympathetic nerve activity was evaluated by telemetric analyses of the low-frequency component of blood pressure (BP) and by plasma epinephrine and norepinephrine levels. The results showed 6 weeks of EA significantly lowered the increased BP effectively, inhibited the enhanced sympathetic nerve activities and attenuated cardiac hypertrophy in 2K1C hypertensive rats. The level of orexin receptor-1 (OX1R) in the rostral ventrolateral medulla (RVLM) after EA treatment was markedly reduced in 2K1C rats, while there was no difference in the RVLM expression of orexin receptor-2 (OX2R) in 2K1C and 2K1C+EA rats. Moreover, the increased pressor and depressor responses to microinjection of orexin A or OX1R antagonist SB408124 into the RVLM of 2K1C rats were significantly blunted by the EA treatment. These findings suggest that BP-lowering effect of EA on renovascular hypertension may be through inhibition of central sympathetic activities and modulation of functional orexin receptors in the RVLM.

## Introduction

Hypertension is the most common risk factor for cardiovascular disease and death. Antihypertensive medication serves as a major therapy for treating hypertension. Despite a number of available treatment options, a substantial part of the hypertensive population has uncontrolled blood pressure (BP) due to non-adherence or intolerance to available antihypertensive agents (Oparil and Schmieder, 2015). Thus, developing an effective treatment for this population becomes an important goal for treating hypertension and its comorbidities. As a non-pharmacological intervention, acupuncture has been used for centuries in treatment of various disorders, including cardiovascular disease (for review, see Cheng et al., 2015; Longhurst and Tjen-A-Looi, 2017). Electroacupuncture (EA) is a more effective way of administering acupuncture, which apply a pulsating electrical current to acupuncture needles for acupoint stimulation. Clinical and experimental studies indicate that low-frequency EA at Quchi (LI11), Neiguan (PC6), and Zusanli (ST36) acupoints has therapeutic and modulatory effects on some types of hypertension (Zhang et al., 2013; Cheng et al., 2015; Li et al., 2016; Tan et al., 2016; Wang et al., 2018). It has been demonstrated that the nervous system, neurotransmitters, and endogenous substances are involved in EA treatment (Zhou and Longhurst, 2012; Xiao et al., 2017). However, the underlying mechanisms for beneficial antihypertensive responses of EA are unclear.

It is well-known that overactive sympathetic nervous system contributes to the development of hypertension (Grassi et al., 2015). The evidences suggest that EA could lower sympathetic activity and significantly inhibit the sympathoexcitatory reflex responses in rats (Ma et al., 2014; Tan et al., 2016; Tjen-A-Looi et al., 2016). The rostral ventrolateral medulla (RVLM) plays a critical role in the integration and generation of central sympathetic drive via projections to sympathetic preganglionic neurons (Guyenet et al., 2018). Inhibition of neuronal function in this nucleus results in large decreases in BP. Thus, neuronal activity in the RVLM is one of the important targets of EA for modulating sympathetic outflow.

Orexins (orexin-A and -B) are neuropeptides produced by neurons primarily located in the lateral hypothalamic area, and their action is mediated by two G-protein coupled receptors termed orexin receptor-1 (OX1R) and orexin receptor-2 (OX2R) (Huber et al., 2018). Orexin efferent projections are widely distributed throughout the brain including the brainstem areas, and orexin receptors are also widespread throughout the brain including RVLM (Shahid et al., 2012b). Accumulating evidences indicate that the orexin system has a crucial role in the regulation of BP and sympathetic outflow, and contributes to several animal models of hypertension (reviewed by Carrive, 2017). Intracisternal- and RVLM-injection of orexin evoke a rise in BP and HR in both anesthetized and conscious rats, with orexin-A being made more effective (Chen et al., 2000; Machado et al., 2002). It was reported that microinjections of orexin-A in the vasopressor region of the RVLM depolarized vasopressor neurons which inhibit vagal activity and increase sympathetic outflow, and these effects can be attenuated by orexin receptor antagonists (Huang et al., 2010; Shahid et al., 2012a; Li et al., 2013). Accordingly, an immunohistochemical study has also revealed that orexin-A immunoreactive neurons in the tuberal hypothalamus project to the medulla (Lee et al., 2015). Several studies have shown that prepro-orexin knockout mice and orexin neuron-ablated mice (orexin-ataxin3 transgene) have a significantly lower BP than wild-type controls (Kayaba et al., 2003; Zhang et al., 2006). An increase of orexin-A expression in the lateral hypothalamus and OX1R in the RVLM has also been reported in stress induced-hypertensive rats, which was accompanied by an increase of BP (Xiao et al., 2013). The data have indicated that RVLM, a key neural structure involved in mediating the sympathetic activity of orexin-A in the brain, plays an important role in regulating BP. However, whether RVLM orexin system is involved in acupuncture treatment of hypertension is unknown.

In the present study, we tested the hypothesis that the antihypertensive and sympathoinhibitory effect of EA was associated with the modulation of increased activity of the orexin system within the RVLM of hypertensive rats.

## Materials and Methods

### Animals

Male Sprague-Dawley (SD) rats with the weight 200 to 220 g were used for this experiment. The rats were purchased from the Laboratory Animal Center of Xi’an Jiaotong University, and housed under controlled conditions with a 12 h:12 h light–dark cycle, 22 ± 2°C room temperature and 50 ± 5% humidity. Food and water were available to the animals ad libitum. All protocols were approved by the Institutional Animal Care and Use Committee of Shaanxi University of Chinese Medicine (No. 2015022).

### Two-Kidney, One Clip (2K1C) Hypertensive Model

Sprague-Dawley rats were randomly divided into four groups after 1 week of adaptation: the sham group (Sham, *n* = 12), sham plus EA treatment group (Sham+EA, *n* = 12), 2K1C hypertensive group (2K1C, *n* = 16), and 2K1C plus EA treatment group (2K1C+EA, *n* = 16). The 2K1C hypertensive model was established as previously described (Zhang et al., 2018). Briefly, the rats were anesthetized with pentobarbital (i.p., 40 mg/kg), and the adequacy of anesthesia was evaluated by the loss of a pedal withdrawal reflex. A retroperitoneal flank incision was performed under sterile surgical condition. The right kidney was exposed and the right renal artery was isolated. The renal artery was partly obstructed with a silver clip with an internal diameter of 0.20 mm. Sham-operated rats were performed the same surgical procedures without clip placement. The kidney was repositioned and the wound was sutured. The rats were kept in cages after surgery, maintained on 12 h light/dark cycle with free access to normal chow and tap water. The model was successfully established if systolic BP exceeds 30 mmHg than pre-operation after 4 weeks of operation (Oliveira-Sales et al., 2016; Carvalho-Galvão et al., 2018). In 41 rats with a clipped renal artery, 32 rats were hypertensive and 9 rats were excluded because their BP was not high enough to meet the criterion at 4 weeks after clipping.

### EA Treatment

Four weeks after 2K1C or sham surgery, the EA treatment was applied as previously described (Tan et al., 2016; Tao et al., 2016; Zhang et al., 2018). Rats were adapted and handled gently for 30 min each day, for 3 days before the beginning of the experiment. The bilateral Quchi (LI11) acupoints were selected for EA application and the positioning of LI11 acupoints in rats was referred to the standard acupoint map in Experimental Acupuncture (Li, 2003). Stainless steel needles (0.16 mm diameter) were inserted bilaterally in the LI11 acupoint, which was located in the midpoint between the lateral end of the transverse cubical crease and the lateral epicondyle of the humerus of the upper limb, and was innervated by the radial nerve. The electrical stimulation was performed using Hans-200A electrostimulator (Beijing Sheng Da Medical Instrument Center, Beijing, China) at alternate frequencies (2 and 15 Hz), with 0.5 ms in duration and certain intensity (≤4 mA) which elicited slight muscle contraction or movement of the paw (Lima et al., 2015; Li et al., 2016). The treatment was applied for 30 min once a day for 6 weeks. In the sham control group and 2K1C group, rats were stayed for a 30-min period with needle insertion but without electrical stimulation of LI11 acupoints. All procedures were performed by the same researcher.

### BP Measurement

Conscious BP recording was carried out with a radiotelemetry system, as described previously (Zhang et al., 2009). Ten days before making a 2K1C surgery or sham operation, the rats were anesthetized with 3% isoflurane inhalation. Under conventional disinfection and sterility conditions, the abdominal aorta was exposed and isolated. The catheter of telemetry BP probe (TA11PA-C40, Data Sciences International, Saint Paul, MN, United States) was inserted into the lower abdominal aorta and the probe was positioned intra-abdominally and secured to the ventral abdominal muscle. The telemetry signals were recorded and processed by using Dataquest IV system (version 4.33, Data Sciences International, Saint Paul, MN, United States), and the average arterial BP and heart rate (HR) for each day were calculated from values recorded during a 3-h period from 9:00 a.m. to 12:00 pm in a conscious state. Continuous recordings were started 4 days after the probe implantation. In addition, Arterial BP waveforms from the radiotelemetry recordings (500 Hz) were used to assess the low versus high frequency components of BPV by spectral analysis. For each animal, approximately 2 h of continuous recordings were imported into an in-house written computer program using MATLAB 2015b (MathWorks, Natick, MA, United States) for calculation of frequency domain measures of autonomic tone. Parameters measured were: low frequency (measure of sympathetic tone) – LF (0.2–0.8 Hz), high frequency (measure of cardiac parasympathetic and potentially respiration) – HF (0.8–2.5 Hz), Total Power – TP (0.2–2.5 Hz), LF/HF (measure of sympathetic/parasympathetic balance), and LF/TP.

Spontaneous baroreflex sensitivity was assessed by the sequence method using the radiotelemetry data as our previously described (Zhang et al., 2018). Analysis was performed with HemoLab Software Ver. 20.5. Baroreflex sequence was identified as the correlation coefficient (r) between systolic BP and pulse interval values greater than or equal to 0.8. Baroreflex sensitivity was calculated from the slope of the linear regression lines between systolic BP and the pulse interval of each baroreflex sequence, and the up-sequence gain (in ms/mmHg), down sequence gain (in ms/mmHg), and overall baroreflex gain (in ms/mmHg) were determined.

For the microinjection studies, acute BP recording was performed using PE50 catheters under isoflurane (3%) anesthesia at the end of the 6-week EA treatment. The PE50 catheters filled with heparin saline (100 IU/mL) was inserted into the left femoral artery, and connected to a BP transducer and bridge amplifier (AD Instrument, Sydney, NSW, Australia). The BP and HR were recorded and analyzed with PowerLab data analysis system (AD Instrument, Sydney, NSW, Australia).

### Microinjection Experiments

At the end of the 6-week EA treatment, the BP and HR data were collected and analyzed with PowerLab software (AD Instrument, Sydney, NSW, Australia). RVLM microinjection was performed according to the procedures described previously (Zhang et al., 2009). In brief, the rat was placed in a stereotaxic frame under isoflurane (3%) anesthesia. After surgical exposure of the dorsal medulla oblongata, a multiple-barrel glass injection pipette (tip size 20–40 μm) connected to a pressure microinjection apparatus was positioned in the RVLM. The coordinates for RVLM were, with the pipette tip angled 20° rostrally, 1.8–2.0 mm lateral from the midline, 1.6–1.8 mm rostral to the caudal tip of area postrema, and 2.8–3.0 mm below the dorsal surface. Proper placement was confirmed by observing changes in BP with microinjections of glutamate (300 pmol, in 50 nL), which induced a characteristically abrupt pressor response (ΔBP > 20 mmHg) (Zhang et al., 2009). Once a responsive site was located, the probe remained in this site throughout the remainder of the experiment. The volume of microinjection was determined by the displacement of fluid meniscus in the micropipette barrel under a microscope. All drugs were dissolved in artificial cerebrospinal fluid and injected in 50 nL over a period of 4 to 6 s. Rat body temperature was maintained in the range of 36.5–37.5°C with a heating pad. After the protocol, microinjection sites were identified with methylene blue dye (50 nL) and confirmed histologically.

### Measurement of Norepinephrine (NE) and Epinephrine (E) Levels

Plasma samples were collected from the abdominal aorta at the end of experiment. The levels of NE and E in plasma were quantified using commercially available rat enzyme-linked immunosorbent assay (ELISA) kits (Rocky Mountain Diagnostics, Colorado Springs, CO, United States) according to manufacturer’s instructions.

### Histological Analysis

At the end of the experiments, rats were euthanized as above, and the hearts and tibiae were dissected and weighed or measured to determine heart weight/tibia length (HW/TL) ratios as described previously (Yin et al., 1982). Hearts were fixed in 10% formalin for 48 h and embedded in paraffin. The middle segments of the hearts were sectioned with a thickness of 10 mm, and the transverse sections were stained with hematoxylin and eosin. Micrographs were detected using a microscope equipped with digital camera (BX53, Olympus, Tokyo, Japan). Approximately 10 visual fields on two randomly selected sections from each animal were visualized using an objective with a calibrated magnification (400×). The histology and morphology were examined and analyzed by two-three blinded researchers using Image Analysis Software (Image Pro plus 6.0, Media Cybernetics, Bethesda, MD, United States).

### Real-Time PCR

The animals were killed with an excessive dose of sodium pentobarbital, and the RVLM tissues were collected. OX1R and OX2R mRNA were analyzed using quantitative real-time PCR as detailed by us previously (Zhang et al., 2009). The total RNA was isolated from RVLM tissue lysates using a RNeasy kit (Qiagen, Valencia, CA, United States) according to the manufacturer’s instructions. TaqMan PCR probes for rat OX1R (Rn00565032_m1), OX2R (Rn00565155_m1), and 18S rRNA (Rn03928990_g1) were obtained from Thermo Fisher Scientific (Carlsbad, CA, United States). Amplification was performed in an Applied Biosystems PRISM 7000 sequence detection system. A comparative cycle of threshold (CT) fluorescence method was used with 18S rRNA as the internal control. The final data were showed as the ratio of the mRNA of interest to 18S rRNA.

### Western Blot

The procedures were as described previously (Zhang et al., 2009). RVLM samples were homogenized in an ice-cold lysing buffer, and the protein concentration was determined by a protein assay kit (Bio-Rad Laboratories, Hercules, CA, United States). The samples were separated by SDS-PAGE gel and transferred electrophoretically to nitrocellulose membranes (Millipore, Billerica, MA, United States). After blocking with 5% skim milk, the membranes then were hybridized at 4°C overnight with the primary antibody, rabbit polyclonal anti OX1R (1:1000, # ab68718, Abcam, Cambridge, United Kingdom) or rabbit polyclonal anti OX2R (1:1000, # ab183072, Abcam, Cambridge, United Kingdom), and further incubated with secondary antibody (goat anti-rabbit IgG horseradish peroxidase, Bio-Rad, 1: 3000). Then the membrane was treated with enhanced chemiluminescence substrate (ECL Western blotting detection kit, Amersham Pharmacia Biotechnology, Piscataway, NJ, United States). The bands in the film were visualized and analyzed using Quantity One Software (Bio-Rad).

### Statistical Analysis

All data are expressed as means ± SE. The distributions of all data were tested for normality using the D’Agostino & Pearson normality test by SigmaStat 3.5 software. Comparisons between experimental groups were performed with ANOVA followed by a Newman–Keuls test. Differences were considered significant at *P <* 0.05.

## Results

### Antihypertensive Effect of EA Treatment in 2K1C Rats

**FIGURE 1:**
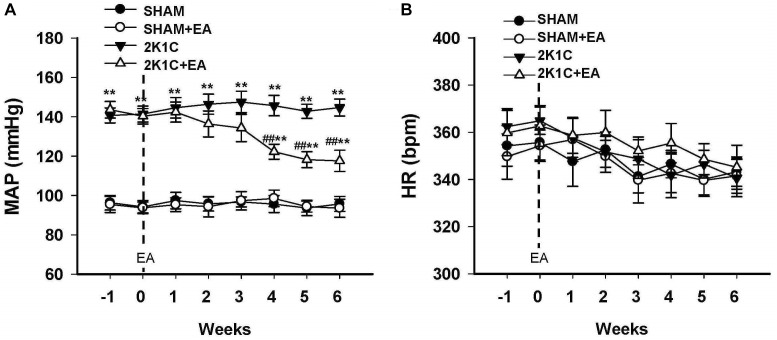
Antihypertensive effect of EA treatment in 2K1C rats. **(A)** Time course of mean arterial pressure (MAP) in each group over 6 weeks. **(B)** Time course of heart rate (HR) in each group over 6 weeks. MAP and HR were measured by radiotelemetry. Data are presented as mean ± SE; *n* = 6–8 rats; *^∗^P <* 0.05, *^∗∗^P <* 0.01 vs. the SHAM group; *#P <* 0.05, *##P <* 0.01 vs. the 2K1C group.

Mean arterial BP (MAP) and HR of rats in sham, sham+EA, 2K1C, and 2K1C + EA groups were recorded by radiotelemetry. As shown in Figure 1A, the MAP was increased in the 2K1C group compared with that in the sham group. After rats were subjected to EA treatment at LI11 acupoint for 6 weeks, rats in the 2K1C+EA group displayed a significantly decreased MAP from weeks 4 to 6 compared with that in the 2K1C group (*P <* 0.01). The rats in the sham + EA group did not show a significant change in the MAP compared to that in the sham group. In addition, no significant alterations in HR were observed in all groups (Figure 1B).

### Effects of EA Treatment on Sympathetic Activities in 2K1C Rats

**FIGURE 2:**
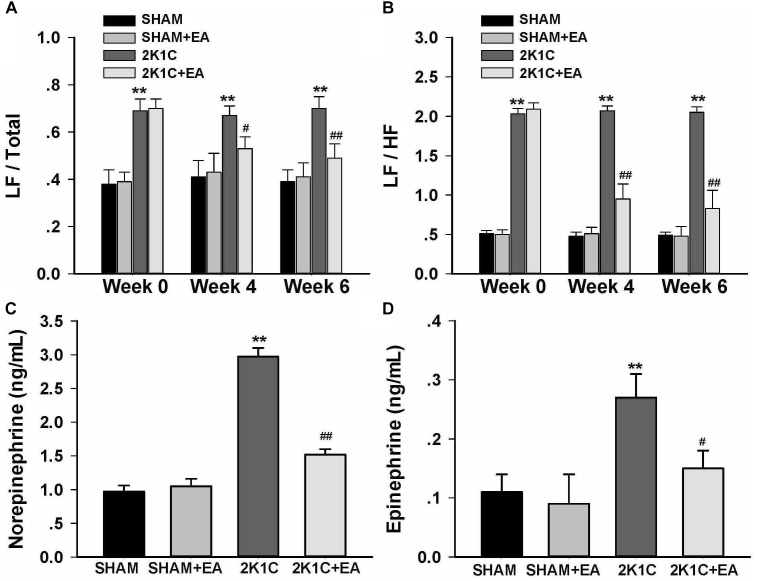
Sympathoinhibitory effect of EA treatment in 2K1C rats. Arterial BP waveforms from the radiotelemetry recordings (500 Hz) were used to assess the low vs. high frequency components of BP variability by spectral analysis before (0 week) and after (4 or 6 weeks) EA or Sham treatment as an index of changes in sympathetic nerve activity. **(A)** Changes of low frequency/total (LF/Total) ratios in each group at baseline, 4 and 6 weeks after EA or Sham treatment. **(B)** Changes of low frequency/high frequency (LF/HF) ratios in each group at baseline, 4 and 6 weeks after EA or Sham treatment. **(C)** Plasma norepinephrine content at 6 weeks after EA or Sham treatment. **(D)** Plasma epinephrine content at 6 weeks after EA or Sham treatment. Data are presented as mean ± SE; *n* = 6–8 rats; *^∗^P <* 0.05, *^∗∗^P <* 0.01 vs. the SHAM group; *#P <* 0.05, *##P <* 0.01 vs. the 2K1C group at same time point.

To determine whether chronic EA treatment altered autonomic nerve activities, changes in the frequency components of BP variability were examined by spectral analysis at baseline, 4, and 6- weeks following EA. As compared to values of sham rats, 2K1C rats displayed a significant and sustained increase in the LF/Total and LF/HF ratios at 0, 4-, and 6-weeks following EA (Figure 2A,B). Long term EA treatment in the 2K1C+EA group produced a significant decrease in the sympathetic component of BP variability at 4 and 6 weeks compared with that in the 2K1C group (Figure 2A,B).

**FIGURE 3:**
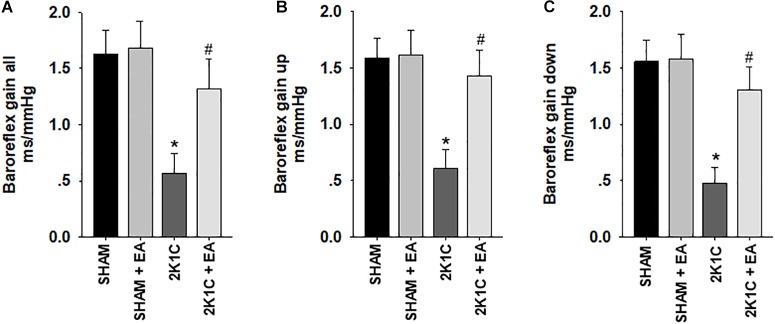
Electroacupuncture (EA) treatment restores the baroreflex response in 2K1C hypertensive rats. Spontaneous baroreflex analysis with overall baroreflex gain (combination of up and down sequences) **(A)**, up sequence gain **(B)**, and down sequence gain **(C)** (in ms/mmHg). Data are presented as mean ± SE; *n* = 7 rats; ^∗^*P* < 0.01 vs. the sham group; #*P* < 0.05 vs. the 2K1C group.

Figure 3 showed that the 2K1C group presented a reduction in baroreflex gain (total gain, up sequence gain, and down sequence gain) compared with the sham group, and the 2K1C +EA group exhibited the restored baroreflex sensitivity compared with the 2K1C group.

The effect of EA treatment on plasma norepinephrine and epinephrine level in 2K1C rats at the completion of the 6-week treatment was shown in Figure 2C,D. As compared to levels in 2K1C rats, both the plasma norepinephrine and epinephrine contents were significantly reduced in 2K1C+EA rats at 6 weeks following EA treatment (*P <* 0.05).

**FIGURE 4:**
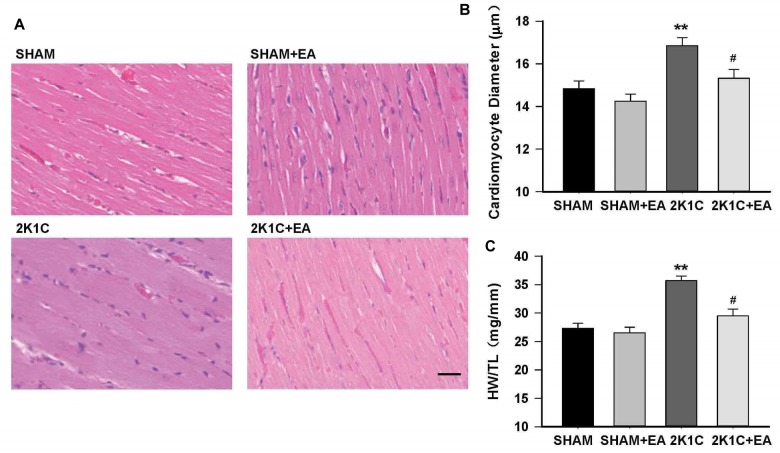
Electroacupuncture treatment protects against cardiac hypertrophy in 2K1C rats. **(A)** HE stained representative images of left ventricular free wall. **(B)** Quantification of cardiomyocyte diameter in each group. **(C)** Heart weight to tibia length (HW/TL) ratio in each group. The scale is 50 μm. Data are presented as mean ± SE; *n* = 6–8 rats; *^∗∗^P <* 0.01 vs. the SHAM group; *#P <* 0.05 vs. the 2K1C group.

In addition, the 2K1C group presented an increase in the cardiomyocyte diameter and the ratio of heart weight to tibia length (HW:TL) compared with the sham group, and the 2K1C +EA group exhibited the improvement in myocardial hypertrophy compared with the 2K1C group (*P <* 0.05, Figure 4).

### Effect of EA Treatment on OX1R and OX2R Expressions in RVLM

**FIGURE 5:**
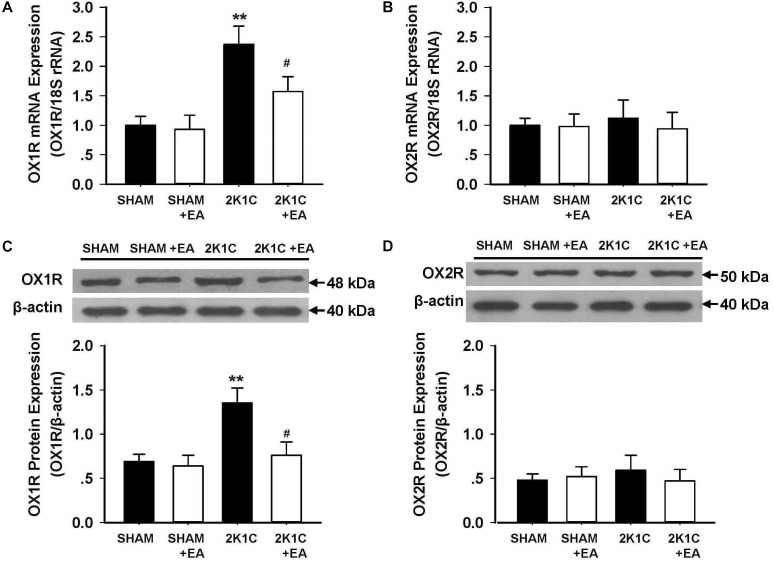
Effect of EA treatment on the expression of orexin receptors in the RVLM of 2K1C rats. **(A,B)** Orexin 1 receptor (OX1R) and Orexin 2 receptor (OX2R) mRNA levels within the RVLM in each group detected with real-time RT-PCR. Data were normalized with 18S rRNA. **(C,D)** Quantitative analysis of OX1R and OX2R protein levels within the RVLM in each group. Upper panel show the representative immunoblots of OX1R and OX2R protein levels. Values are normalized using β-actin. Values are means ± SE (*n* = 6 in each group). *^∗∗^P <* 0.01 vs. the SHAM group; *#P <* 0.05 vs. the 2K1C group.

The expression of the orexin system was examined in the RVLM in each group. Figure 5A,B show the mRNA changes of OX1R and OX2R in RVLM of 2K1C rats after 6 weeks of EA stimulation. OX1R mRNA levels in the RVLM increased in the 2K1C group, and EA attenuated this change. No differences were observed in OX2R mRNA expression in the RVLM for all groups. The immunoblotting analysis, as shown in Figure 5C,D, demonstrated that the 2K1C group presented a twofold increase in OX1R protein levels compared with the sham group, and EA treatment significantly reversed the expression changes of OX1R in the 2K1C +EA group when compared to the 2K1C group. In contrast, the expression of OX2R was comparable between the groups. We also examined the change of orexin-A level in RVLM of each group by ELISA method, and the results showed no significant difference in orexin-A level in RVLM among the SHAM, SHAM+EA, 2K1C, and 2K1C+EA groups (*P* > 0.05, Data are not shown).

### Effect of EA Treatment on OX1R-Mediated Responses in RVLM

**FIGURE 6:**
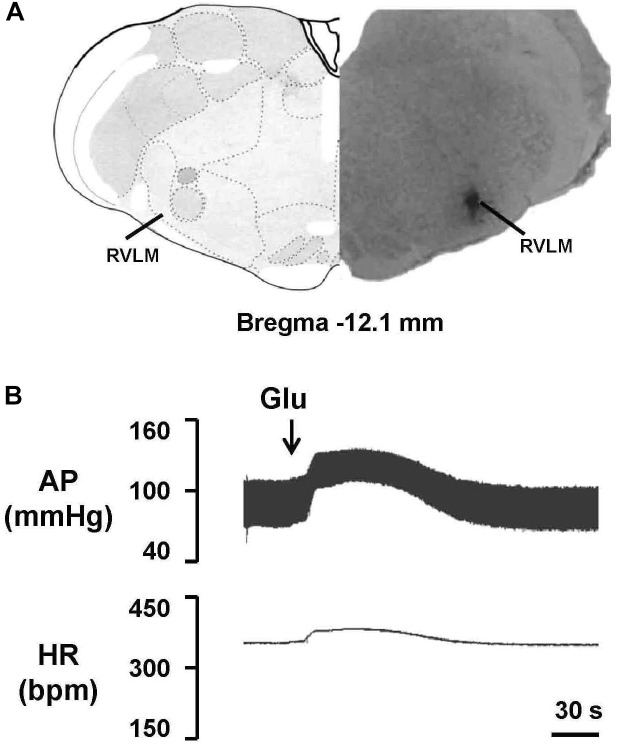
Identification of the microinjection sites in the rostral ventrolateral medulla (RVLM). **(A)** A representative photomicrograph showing the RVLM microinjection site, and the location of this microinjection site based on the rat brain atlas. Arrow indicates the injection site. **(B)** Functional identification of the RVLM with microinjections of glutamate (Glu; 300 pmol in 50 nl) in one rat. Unilateral glutamate produced increases in arterial pressure (AP) and HR.

**FIGURE 7:**
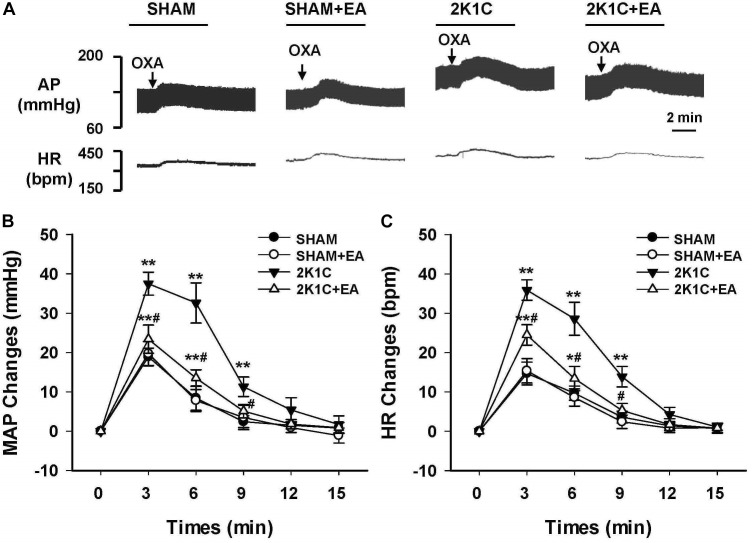
Changes of mean arterial pressure (MAP) and heart rate (HR) after microinjection of orexin A in the RVLM of rats. **(A)** Representative original tracings showing AP and HR changes evoked by microinjection of Orexin A (50 pmol in 50 nl) into the RVLM in the SHAM, SHAM+EA, 2K1C, and 2K1C+EA groups. Horizontal bar represents recording duration of 2 min. Arrows indicates the injection of orexin A (OXA). Time course of MAP **(B)** and HR **(C)** changes in each group over 15 min. Values are means ± SE (*n* = 6–8 in each group). *^∗^P <* 0.05, *^∗∗^P <* 0.01 vs. pre-injection control (time 0); *#P <* 0.05 vs. 2K1C group at same time point.

To evaluate the effect of EA treatment on OX1R function in the RVLM, orexin A or OX1R antagonist SB408124 was microinjected into the RVLM of rats at the completion of the 6-week treatment. BP and HR were recorded before and after microinjection, as described in METHODS. Figure 6 shows the microinjection sites in the RVLM which were identified with microinjections of glutamate. Figure 7 shows the time course of MAP and HR before and after microinjection of orexin A (50 pmol, 50 nL) into the RVLM of rats. MAP and HR were increased in varying degrees in different groups following orexin A microinjection into the RVLM. In the 2K1C group, orexin A induced a robust increase of MAP and HR at 1 min post-injection, and this pressor effect lasted about 9 min. In contrast, orexin A induced a smaller pressor response in RVLM of sham rats, and this response lasted only about 6 min. The response to orexin A was greater in the 2K1C group when compared with that in the sham group. Treatment with EA for 6 weeks normalized the response to orexin A in 2K1C rats (Figure 7B). Changes in HR after microinjection of orexin A into the RVLM followed the same pattern as MAP (Figure 7C).

**FIGURE 8:**
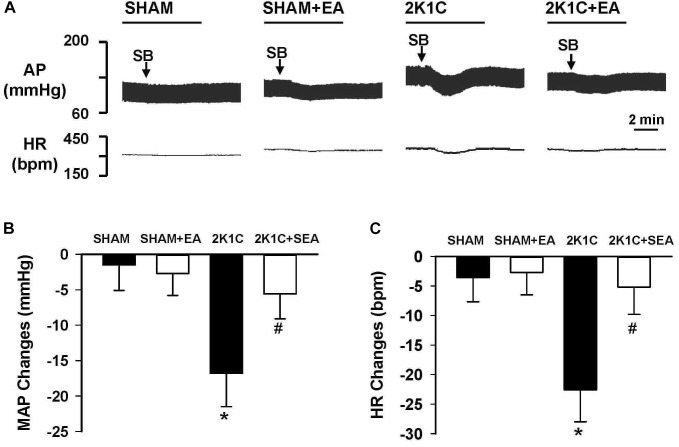
Changes of mean arterial pressure (MAP) and heart rate (HR) by microinjection of Orexin 1 receptor (OX1R) antagonist, SB408124, in the RVLM of rats. **(A)** Representative original tracings showing AP and HR changes evoked by microinjection of SB408124 (100 pmol in 50 nl) into the RVLM in the SHAM, SHAM+EA, 2K1C, and 2K1C+EA groups. Horizontal bar represents recording duration of 2 min. Arrows indicates the injection of SB408124 (SB). **(B)** The peak alteration of MAP after microinjection of SB408124 (100 pmol in 50 nl) into the RVLM in the SHAM, SHAM+EA, 2K1C, and 2K1C+EA groups. **(C)** The peak alteration of HR after microinjection of SB408124 (100 pmol in 50 nl) into the RVML in the SHAM, SHAM+EA, 2K1C, and 2K1C+EA groups. Values are means ± SE (*n* = 5–7 in each group). *^∗^P <* 0.05 vs. the SHAM group; *#P <* 0.05 vs. the 2K1C group.

As shown in Figure 8, peak alteration of MAP and HR were analyzed after microinjection of OX1R antagonist SB408124 (100 pmol in 50 nl) into the RVLM of rats in investigated groups. The amplitudes of MAP change induced by SB408124 in the 2K1C group were significantly greater than those in the sham group. In contrast, treatment with EA for 6 weeks significantly reduced the MAP change in the 2K1C + EA group when compared with the 2K1C group. Changes in HR after microinjection of SB408124 into RVLM had the same pattern as MAP (Figure 8C).

## Discussion

High BP is a common and dangerous condition. Clinical and animal studies have revealed that acupuncture at certain acupoints is effective in reducing arterial BP and end-organ damage in hypertension and has been recommended as a non-pharmacological therapy (Lee et al., 2009; Wang et al., 2013; Longhurst and Tjen-A-Looi, 2017). In the present study, EA treatment at LI11 acupoints was applied for 6 weeks in 2K1C hypertensive rats, and the results demonstrated that (1) EA treatment effectively reduced arterial BP and decreased the enhanced sympathetic activities in 2K1C rats, (2) EA treatment significantly improved the myocardial hypertrophy, and (3) EA treatment inhibited the increased OX1R expression and reversed the OX1R-mediated responses in the RVLM of the hypertensive rat. These results suggest that the anti-hypertensive effect of EA treatment was related to the modulation of functional OX1R in the RVLM and inhibition of abnormal sympathetic nerve activities.

Acupuncture of the LI11 acupoints overlying the deep radial nerves has been widely used in treating cardiovascular diseases by the traditional Chinese Medicine (Litscher et al., 2014; Chavez et al., 2017). It has been demonstrated that EA at the LI11 is most effective in its influence on cardiovascular excitatory reflexes induced by gastric distension and gall bladder stimulation (Tjen-A-Looi et al., 2004). We have also shown that EA at LI11 resulted in significant reductions in elevated BP of hypertensive rats (Tan et al., 2016). Although EA at LI11 ameliorate BP in patients with mild to moderate hypertension, the underlying mechanisms are unknown. In this study, we used a telemetric system to investigate the effects of EA on conscious BP, HR, and BP variability in 2K1C rats. Continuous recordings of BP in 2K1C rats treated with EA stimulation for 6 weeks demonstrated that the EA at LI11 had a long-term BP-lowering effect, which supports previous clinical findings. Notably, no significant changes in arterial BP were observed in control normotensive rats given the same pattern of EA treatment. These observations are consistent with the results reported by Huo et al. (2014), which states that EA reduce the elevated arterial BP in spontaneously hypertensive rats but has no significant effect on Wistar rats. Studies in healthy subjects also demonstrated that EA applied to healthy subjects at rest does not change BP or heart rate (Li et al., 2004). We speculated that acupuncture has the effect of correcting the dysfunctions of BP control signals (such as abnormal sympathetic activity) without affecting physiological regulation signals, which may be the advantage of acupuncture over other strategies (Tjen-A-Looi et al., 2003).

Enhanced sympathetic activity has been strongly implicated in the development and maintenance of hypertension (Grassi et al., 2015). Accumulating evidence indicated that hypertension in 2K1C rats is associated with enhancements of sympathetic activity (Oliveira-Sales et al., 2016; Li et al., 2017). To determine the potential role of the autonomic nervous system in the BP-lowering effect of EA treatment, we analyzed the LF of BP and LF/HF ratio by spectral analyses of the telemetric data. We found that the LF/Total and the LF/HF ratio in the 2K1C+EA group were significantly less than that of the 2K1C group during both 4w and 6w EA treatment. Furthermore, plasma norepinephrine and epinephrine levels in the 2K1C+EA group were significantly lower than those in the 2K1C group after EA treatment. These findings demonstrate that EA inhibits sympathetic activities in 2K1C rats. Thus, the sustained BP-lowering effect and reduction in BP variability that were induced by EA treatment, at least partially, were mediated by improvements in the autonomic nervous function.

The RVLM is one of the major brain regions for control of basal and reflex sympathetic activity. The bulbospinal neurons of RVLM integrate and develop the central sympathetic outflow via projections to sympathetic preganglionic neurons in the spinal cord (Guyenet et al., 2018). Increasing evidence indicates that the RVLM is also involved in the conditions of augmented sympathetic activities associated with cardiovascular diseases (Guyenet, 2006; Mischel et al., 2015). It has also been demonstrated that the neuropeptides and transmitters in RVLM play a long-term regulatory effect on neurons excitability which may affect adaptive reflexes and encode distinct patterns of sympathetic output, and an overactive orexin system was implicated in the enhanced sympathetic vasomotor tone in the hypertension pathogenesis (Miyawaki et al., 2002; Pilowsky et al., 2008). Consistent with previous studies (Li et al., 2013; Xiao et al., 2013), we found that the expression of orexin receptor subtype OX1R were significantly higher in the RVLM of 2K1C rats than that in sham rats, and the OX2R levels in RVLM had no significant change between 2K1C and sham rats. Furthermore, we found that the increases in MAP and HR induced by microinjection of orexin A into the RVLM were significantly greater in 2K1C than in sham rats. These observations suggest that one source of the enhanced sympathetic tone may be mediated by orexin neurons in the RVLM of hypertensive rats. We also observed the effect of EA treatment on RVLM orexin system, and the results indicated that 6-week EA treatment at LI11 attenuated OX1R expression and function in the RVLM of 2K1C rats. This outcome underlies that the inhibiting orexin receptors in the RVLM might be involved in the antihypertensive effect associated with long term EA application.

Although orexin-containing neurons primarily located in the lateral hypothalamic area, orexin projections and orexin receptors are widely distributed in the brain sites including the RVLM and nucleus tractus solitarius (Shahid et al., 2012b; Huber et al., 2018). In current study, blocking OX1R with SB408124 in the RVLM reduced MAP and HR in 2K1C rats did not alter basal BP and HR in sham normotensive rats. These data are consistent with the previous results observed in the RVLM of spontaneous hypertensive rats (Li et al., 2013), which suggests that OX1R is tonically activated in the RVLM to support the elevated sympathetic activities in hypertension. Tonic activation of OX1R could be due to upregulation of OX1R expression or increases in the orexin levels in the RVLM in hypertension (Zhou et al., 2015). However, orexin A levels in the RVLM are not significantly different between 2K1C and sham normotensive rats. Thus, these data indicate that upregulation of OX1R function and expression in RVLM is associated with hypertension.

One concern is the possible mechanism of acupuncture intervention on the activity of RVLM neurons. Acupuncture needles are typically inserted at acupoints located along meridians. It has been suggested that neural pathways located beneath meridians are responsible for the action of acupuncture, and particularly deep somatic sensory nerve (Li and Longhurst, 2010). Stimulation at specific acupoints activates underlying sensory neural pathways that project to a number of regions in the central nervous system that ultimately regulate autonomic outflow and hence cardiovascular function (Li and Longhurst, 2010; Choi et al., 2012). Electrophysiological studies have shown that sensory stimulation of the deep somatic afferent modifies neuronal activities in the RVLM, and resection of the deep radial nerves eliminates the therapeutic effect of EA (Tjen-A-Looi et al., 2004; Zhou et al., 2005). However, the precise route by which sensory signals elicited from acupuncture transits the RVLM to activate outgoing regulation signals needs further investigation.

In summary, our data demonstrate that inhibition orexin system in the RVLM contributes to antihypertensive and sympathoinhibitory action of EA in rats with sustained hypertension. Our findings provide further evidence and insight into the beneficial effect of EA on hypertension.

## Data Availability

The datasets generated during and/or analyzed during the current study are available from the corresponding author on reasonable request.

## Author Contributions

QZ and Y-YT designed the study, analyzed the data, and wrote the manuscript. Y-YT, LF, and QZ performed the experiments and data collection. F-RY and D-YC provided critical technical assistance and revised the manuscript. All authors approved the manuscript.

## Conflict of Interest Statement

The authors declare that the research was conducted in the absence of any commercial or financial relationships that could be construed as a potential conflict of interest.
